# Supplementary material for: Exploring the reasons for novice nurse educators’ transition from practice to academia in Ghana
Source: PLoS One. 2021 Oct 14;16(10):e0258695. doi: 10.1371/journal.pone.0258695 (PMC8516293; doi:10.1371/journal.pone.0258695)
Supplement: S1 Table — (DOCX) [file pone.0258695.s001.docx]

**Table 2. Demographic characteristics of participants**

| **Code** | **Age** | **Sex** | **Marital status** | **Highest degree attained** | **Years in clinical practice** | **Years in nursing academia** |
| --- | --- | --- | --- | --- | --- | --- |
| NNE1 | 33 | M | Married | BSc. Nursing | 7 | 2 |
| NNE2 | 39 | M | Married | BSc. Nursing | 10 | 2 |
| NNE3 | 33 | M | Married | BSc. Nursing | 6 | 1 |
| NNE4 | 34 | M | Married | BSc. Public Health | 7 | 2 |
| NNE5 | 32 | F | Married | BSc. Midwifery | 6 | 2 |
| NNE6 | 32 | M | Married | BSc. Nursing | 6 | 2 |
| NNE7 | 35 | M | Married | BSc. Nursing | 8 | 2 |
| NNE8 | 36 | M | Married | BSc. Nursing | 8 | 1 |
| NNE9 | 34 | M | Married | BSc. Nursing | 7 | 2 |
| NNE10 | 37 | M | Married | BSc. Nursing | 9 | 2 |
| NNE11 | 35 | F | Married | BSc. Public Health | 7 | 1 |
| NNE12 | 33 | M | Married | BSc. Nursing | 6 | 2 |
