# Supplementary material for: Exploring the reasons for novice nurse educators’ transition from practice to academia in Ghana
Source: PLoS One. 2021 Oct 14;16(10):e0258695. doi: 10.1371/journal.pone.0258695 (PMC8516293; doi:10.1371/journal.pone.0258695)
Supplement: S2 File — (DOCX) [file pone.0258695.s003.docx]

**Semi-structured interview guide**

**Section A: Demographic data Code………………...**

Age of participant…………………………………………………………………………………...

Gender of participant………………………………………………………………………………..

Marital status………………………………………………………………………………………..

Highest degree obtained…………………………………………………………………………….

Formal education in teaching……………………………………………………………………….

Number of years in clinical practice………………………………………………………………...

Number of years in academia……………………………………………………………………….

**Section B: Reasons for transitioning from practice to academia**

1. Please tell me the reason why you left clinical nursing practice?

- How flexible was your role at the clinical setting?
- Describe how satisfied you were with clinical practice?

1. What were your expectations before moving to nursing academia?

- How did these expectations influence you to move into nursing academia?
- To what extent have your expectations been met?

1. What factors attracted you to nursing academia?

- What informed your decision to become a nurse educator?
- What clinical experiences influenced you to move into academia?

1. Do you see yourself continuing to work in nursing academia; If yes, why? If no, why?
